# Assessment of Potential Benefits of Functional Food Characteristics of Beetroot Energy Drink and Flavored Milk

**DOI:** 10.1155/2022/1971018

**Published:** 2022-03-15

**Authors:** Seema Ashraf, Syed Asad Sayeed, Rashida Ali, Fahim Vohra, Naseer Ahmed, Mohammad Khursheed Alam

**Affiliations:** ^1^Department of Food Science and Technology, Jinnah University for Women, Karachi 74600, Pakistan; ^2^Department of Food Science and Technology, University of Karachi, 75270, Pakistan; ^3^Division of Food Research, HEJ Research Institute of Chemistry, University of Karachi, Karachi 75270, Pakistan; ^4^Department of Prosthetic Dental Science, College of Dentistry, King Saud University, Riyadh, Saudi Arabia; ^5^Department of Prosthodontics, Altamash Institute of Dental Medicine, Karachi 75500, Pakistan; ^6^Department of Preventive Dentistry, College of Dentistry, Jouf University, Sakaka, Al Jouf 72345, Saudi Arabia; ^7^Center for Transdisciplinary Research (CFTR), Saveetha Dental College, Saveetha Institute of Medical and Technical Sciences, Saveetha University, Chennai 600077, India; ^8^Department of Public Health, Faculty of Allied Health Sciences, Daffodil International University, Dhaka 1207, Bangladesh

## Abstract

**Objective:**

This study was designed to determine the antioxidant activity of the extracts of beetroot by using beetroot energy drink and flavored milk (products). *Material & Methods*. This experimental trial was conducted at Jinnah University for Women, Pakistan, under the approval of local institutional review board number JUW/DFST/RCB010/2020. All the materials such as beetroot, carrot, cucumber, and lemon were obtained commercially from which two products were formulated: beetroot energy drink (sample1) and flavored milk (sample 2). These formulated products were evaluated for quality analysis (pH and brix), phytochemical screening using the Keller-Kiliani test, Salkowski's test, Alkaline reagent test, lead acetate test, ferric chloride test, protein test, quantitative test of phenol, antioxidant activity, sensory analysis, and shelf life study. The paired *t*-test was applied to detect significant differences between two samples.

**Results:**

The phytochemical analysis revealed that cardiac glycosides, phytosterol, flavonoids, and terpenoids were found in both energy booster drink (EBD) and flavored milk (FM) except phenolic compounds that were found only in EBD. The antioxidant capacity of beetroot juice was far greater than FM. The statistical sensorial analysis of FM and EBD reported a significant mean difference between most of the groups with *p* < 0.0001.

**Conclusion:**

This study concludes that energy drinks having beetroot indicated higher antioxidant capacity than flavored milk. The nutraceutical products (energy booster drink and flavored milk) containing beetroot are enriched with optimum quantities of proteins and fats and low carbohydrates at a stable pH with an adequate total energy content.

## 1. Introduction

In the past few years, the demands for a healthier diet (foods and beverages) have risen in many countries, where functional foods have caused a disruption in between pharmacotherapy and nutrition [1]. Functional foods are recognized to have health-related stuffs as it contains traditional nutrients. Functional foods specify physiological advantages and lead to lessen the threat of chronic infections due to their nutritious functions, as well as management of gut health [2]. It has been observed that as high as 20% health care expenditure is carried out by the consumption of functional foods only [3].

In 2008, Arai et al. gave an idea for the use of functional foods and assessed the connection between fortification, sensual gratification, nutrition, and physiological system for modulation [4]. Constituents of functional drinks contain necessary fatty acids, minerals, vitamins, herbs, and amino acids. Functional drinks might be beneficial in supporting the immune system, improving the health of the cardiovascular system, weight control, or using as an adjuvant in stabilizing the processes of aging [5].

Functional foods are normally found in almost every product, and the use of energy drinks and flavored milk (beverages) has increased [6]. Beetroot (*Beta vulgaris* L.) is known as an herbaceous biennial plant which is classified as belonging to the Chenopodiaceae family. Beetroot pulp is found as yellow or red in color. Red root is used as a juice extract, in salads, as food color, and also as a medicine [7]. Beets are regarded as very effective vegetable containing anti-inflammatory and antioxidant agents that help in scavenging free radicals from cells that prevent cancer through inhibition of tumor cell proliferation [8]. They are also considered to decrease the risk of cardiovascular diseases as well as expel renal stones [9]. It is suggested that beetroot can reduce as high as 50% oxidation of low-density lipoprotein (LDL) and decrease blood glucose levels by 40% [10]. In addition, beetroot is also a good source of calcium, iron, phosphorus, zinc, sodium, and potassium, in addition to small amounts of biotin, foliate, and niacin vitamins [11]. It is the vegetable in which betalains are found, which are recognized as a highly bioactive pigment group [12, 13]. By heating beetroot, loss and degradation of the essential vitamins and minerals can occur, causing substantial decrease in the health benefits of beetroot. Therefore, fermenting or extracting its juice and mixing with other beverages can help enhance its flavor and retain its biological properties [14, 15]. The fermented beverages containing beetroot are reported to reduce pathogenicity of microorganisms and support in building immunity and improve memory impairment [16]. Some present investigations have revealed that consumption of beetroot gives advantageous physiological outcomes in the disease's atherosclerosis, hypertension, and type-II diabetes mellitus [17–19].

The implication of the present study was to evaluate the characteristics of functional food added in the energy drink as well as in flavored milk in order to improve the physical fitness and mental well-being.

Therefore, the objective of this study was to synthesize beetroot (*Beta vulgaris*) extract energy booster drink (EBD) and flavored milk followed by the analysis of physicochemical properties and phytochemical constituents.

## 2. Material and Methods

This experimental trial was conducted at Jinnah University for Women, Pakistan, under the permission of local institutional review board number JUW/DFST/RCB-010/2020. A total of 40 batches were prepared in the present study, and all the batches further divided into two different samples. Sample 1 consists of 20 batches containing beetroot energy booster drink whereas sample 2 consists of 20 batches containing beetroot-flavored milk.

All the materials such as beetroot, carrot, cucumber, and lemon were obtained commercially and subsequently sorted and categorized. The materials were washed with high-pressure water and treated for extraction. The crude juice was extracted from beetroot by pressing followed by straining to separate undesired remainders.

### 2.1. Preparation of Booster Drink

**Figure 1:**
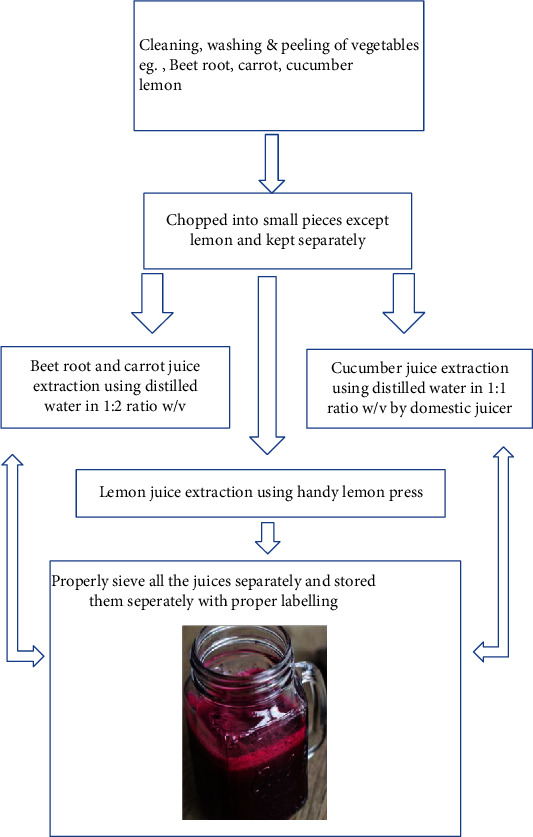
Flow scheme and preparation of energy booster drink.

One liter of juice contained beetroot crude juice (350 ml), carrot crude juice (350 ml), cucumber crude juice (87.5 ml), lemon crude juice (87.5 ml), water for dilution (125 ml), sugar (30 gm), and salt (1 tsp). 20 batches of 1 liter each was prepared and stored in screw-capped glass bottles following mixing of all the above ingredients. All batches were pasteurized at 95–98°C for 15–20 seconds in order to attain retardation of enzymatic action and microbiological safety. Then, liquid juice was transformed into powder by applying the spray technique, and finally, the extract was bottled (Figure 1).

### 2.2. Preparation of Flavored Milk

The standardized and preheated milk (5part) was added into pure beetroot juice (1 part) and sugar (30 g). Ingredients were mixed thoroughly, and milk was pasteurized at 72 ± 2°C for 15–20 seconds. 20 batches of 1 liter were packed in sterilized glass bottles and stored at a suitable temperature as per the analysis requirement.

### 2.3. Qualitative Assessments

Three samples from the two batches (beetroot-flavored milk and energy booster drink (EBD)) were selected randomly. The selected products were evaluated for quality analysis (pH and brix), phytochemical screening, protein test, quantitative test of phenol, antioxidant, sensory analysis, and shelf life study.

#### 2.3.1. Brix and pH Determination

The sample pH was determined to evaluate the acidity of the products by using a pH meter, following standards of the geotechnical test method. The electrode of the pH meter was first cleaned with distilled water and then dipped into the solution (distilled water; 1 : 10 ratio), and after recording the pH, the electrode was cleaned again with distilled water [20]. The refractometric method for the determination of the soluble solid brix was assessed by ISO 2173 : 2003 on ABBE Digital Refractometer [21].

#### 2.3.2. Phytochemical Screening

The phytochemical screening was carried out in order to demonstrate the presence of different types of phytochemicals in beetroot extract. Predefined procedures presented in standard literature were applied [22, 23]. Phytochemical screening was determined by Agbafor and Nwachukwu [24] and Salkowski's test [25, 26]. Numerous qualitative screening techniques were applied, for example, flavonoid was determined by the alkaline reagent test, lead acetate test, and ferric chloride test.

Salkowski's test was used to identify terpenoid. The phenolic compounds were determined by the lead acetate test and ferric chloride test.

#### 2.3.3. Cardiac Glycosides (Keller-Kiliani test)

The Keller-Kiliani test was employed to detect the cardiac glycosides. In this test, 2 ml of test samples was added with 2 ml of glacial acetic acid, one drop of ferric chloride solution, and 1 ml of concentrated sulfuric acid. The formation of a brown ring specified the existence of deoxy-sugar that was verified by the presence of a violet ring [26].

#### 2.3.4. Phytosterol (Salkowski's Test)

Phytosterol was determined by Salkowski's test. 1 ml of sample solution was dissolved in 5 ml chloroform, and few drops of concentrated sulfuric acid were added, and the solution was allowed to stand. The formation of a red ring indicated the presence of phytosterols [26].

#### 2.3.5. Flavonoids

*(1) Ferric Chloride Test*. Determination of flavonoids was carried out by using the method proposed by Asirvatham [27]. A few drops of 1% ferric chloride solution were added in test samples. The intense green color indicated the presence of flavonoids [28]. Flavonoids were also determined by using the lead acetate test, a method proposed by Sindhu and Arora [28], treating samples with few drops of lead acetate. Yellow precipitates indicated the presence of flavonoids. Lastly, the alkaline reagent test was also used for flavonoid assessment. Samples were treated with few drops of sodium hydroxide solution and form yellow color, but this yellow color disappears after the addition of dilute acid which indicates the presence of flavonoids.

#### 2.3.6. Terpenoids (Salkowski's Test)

Five ml of test samples was taken in a test tube, added 5 ml chloroform, and left for evaporating till dry, and then, 5 ml H_2_SO_4_ (conc.) was added followed by heating for 2 minutes. On observation, development of a greyish color indicated the presence of terpenoids [29].

#### 2.3.7. Phenolic Compound

Phenols were determined using the ferric chloride test [27]. 3–4 drops of 1% ferric chloride were added, and the formation of a bluish-black color indicated the presence of phenols. In addition, the lead acetate test was also employed for phenol assessment. The methods proposed by Harborne [30] included the addition of 10% lead acetate in the extract sample, and the formation of bulky-white precipitations indicated the presence of phenolic compounds.

### 2.4. Quantitative Analysis

The test solution was prepared in a 1 : 10 ratio. 1 g of the extracted sample was mixed with 10 ml methanol in a tube, centrifuged for 10 minutes, and shook constantly for a few minutes and then left for 24 hours [31].

#### 2.4.1. Determination of Phenolic Compound

Phenols were determined using the ferric chloride test [27]. 3–4 drops of 1% ferric chloride were added, and the formation of the bluish-black color indicated the presence of phenols. In addition, the lead acetate test was also employed for phenol assessment. The methods proposed by Gibbs [31] included the addition of 10% lead acetate in the extract sample, and formation of bulky-white precipitations indicated the presence of phenolic compounds.

The nitrogen content was determined using the Kjeldahl method in order to calculate the protein content. The protein content was estimated as the nitrogen content of the sample multiplied by a conversion factor. The sample is consumed in sulfuric acid, using CuSO4/TiO2 as catalysts, converting “N” to “NH3,” which is distilled and titrated [32].

(1) $$\begin{array}{c} \%\text{nitrogen}=\frac{\left[\left(\text{ml std}.\text{acid}\times \text{N of acid}\right)-\left(\text{ml blank}\times \text{N of base}\right)\right]-\left(\text{ml std}.\text{base}\times \text{N of base}\right)\times 1.4007}{\text{weight of sample in grams}}. \end{array}$$

#### 2.4.2. Total Antioxidant Activity of Energy Booster Drink (EBD) & Flavored Milk

To detect the antioxidant activity, 2,2-diphenyl-1-picryl-hydrezyl (DPPH) was used as free radical. It was determined through preparation of the sample by a 1 : 10 ratio. 1 g sample (may be powder or liquid) in 10 ml methanol and centrifuged for 10 minutes and the sample was filtered. Filtrate was used for further processing [24, 33].

(2) $$\begin{array}{c} \text{Inhibition percentage}=\text{abs}.\left(\text{DPPH}\right)–\text{abs}.\left(\frac{\text{extracted sample}}{\text{abs}.\left(\text{DPPH}\right)}\right)\times 100. \end{array}$$

#### 2.4.3. Crude Fiber Analysis

Crude fiber was determined by the method proposed in [34]. The sulfuric acid (HSO_4_) solution: 6.25 ml was added in 500 ml distilled water. Sodium hydroxide solution: 6.25 ml NaOH was added in 500 ml distilled water.

1.25 g of dry extract of the beetroot sample was taken, and 500 ml H_2_SO_4_ solution was added in it. It was boiled the mixture for 15 minutes, and then, the solution was filtered through filtration assembly; after that, the filtrate was mixed with 500 ml NaOH solution and the mixture was boiled again for 15 minutes. Lastly, the solution was washed with hot water using filtration assembly weighed and kept in an oven till drying and the residual dehydrated crude fibers were then weighed again.

(3) $$\begin{array}{c} \text{Crude fiber}\%=\frac{100\left(W_{1}-W_{2}\right)}{W}, \end{array}$$

where *W*_1_ is the weight of the sample before drying, *W*_2_ is the weight of the sample after drying, and *W* is the total weight of the sample.

#### 2.4.4. Microbial Quality of the Sample by Standard Plate Count Technique

The microbiological tests, i.e., salmonella, total viable plate count, and aflatoxin tests, were analyzed by following the parameters and guidelines available in standards (PS) [35, 36], for fruit beverages, drinks, and crushes.

#### 2.4.5. Product Shelf Life Study

Both the extracted products were kept at different temperatures and in different packing materials. A brix and pH variation were evaluated as basic taste characteristics to observe the change of quality of the product and shelf life stability on storage.

#### 2.4.6. Sensory Evaluation of Energy Booster Drink and Flavored Milk

The acceptability of the formulated product was assessed by 05 untrained and semitrained sensory panelists who investigated descriptive analysis for energy booster drink and flavored milk from trial 1 to trial 5 of each group. A questionnaire was prepared to assess the sensory attributes of the product. A 9-point hedonic scale (1 = dislike extremely, 9 = like extremely) was used in the questionnaire to evaluate five sensory attributes like color, appearance, taste, aroma, and overall acceptability. Before evaluation, the panelists were demonstrated about the protocol of sensory attributes. Initially, water was used to rinse the mouth between tasting samples; provided an isolated environment, test samples were given them with a sensory score sheet to record their preferences [37].

### 2.5. Statistical Analysis

The particular sensory pro forma was used to collect data. The data was entered in SPSS version 25, for statistical analysis. The paired *t*-test was applied to detect significant differences between two samples. The *p* value of ≤0.05 was considered statistically significant.

## 3. Results

### 3.1. Phytochemical Analysis

With regard to the phytochemical screening of energy booster drink (EBD) and flavored milk (FM), cardiac glycosides were present in good amount in sample 1 while present in moderate amount in sample 2. Phytosterol was present in strong amount in sample 1 and present in moderate amount in sample 2. Phenolic compounds were present in good amount in sample 1 while totally absent in sample 2. For sample 1, flavonoids were present in good amount tested through alkaline reagent and the lead acetate test, while the ferric chloride test showed a moderate amount (sample 1). Sample 2 showed a good number of flavonoids in all the tests. Terpenoids were present in moderate and good amounts in sample 1 and sample 2, respectively, Table 1.

### 3.2. Qualitative Analysis

In terms of the qualitative composition of EBD, the total compositions of carbohydrates, fats, proteins, and fiber were 297.5 g/1000 ml, 10.95 g/1000 ml, 40.25 g/1000 ml, and 29.2 ± 0.009, respectively. The total energy present in the EBD was 352.27 kcal (Table 2). Regarding the composition of flavored drink, fats were 2.56%, proteins (FM) were 4.94%, proteins (juice) were 40.25 ± 0.09, lactose was 7.44%, solid not fat (SNF) was 13.4%, water was 98.3%, its density was 49.3%, and creaming index was 19% while the phosphatase test was negative. The total energy of flavored drink was 72.57 kcal. The total viable plate count (TVPC) for salmonella was absent at <10000 cfu/g as well as for <50000 cfu/g. Melamine was 2.0 ppm at <10000 cfu/g while 2.5 ppm at <50,000 cfu/g. Aflatoxin (M_1_) was at 0.2 ppb in <10000 cfu/g while at 0.5 ppb in <50000 cfu/g (Table 3).

**Figure 2:**
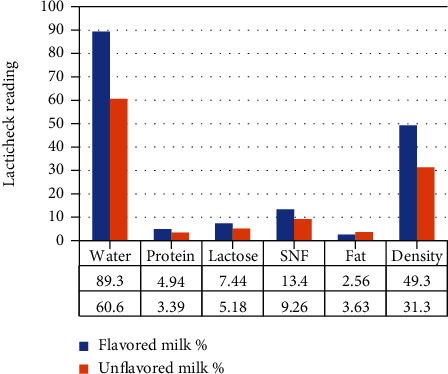
Proximate composition of flavored & unflavored milk.

The proximate composition (LactiCheck reading) in flavored versus nonflavored milk was as follows: water 89.3% versus 60.6%, protein 4.94% versus 3.39%, lactose 7.44% versus 5.18%, solid not fat (SNF) 13.4% versus 9.26%, fat 2.56% versus 3.63%, and density 49.3% versus 31.3% [Figure 2].

**Figure 3:**
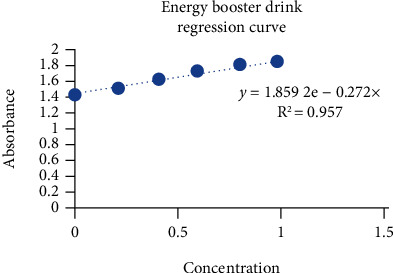
Antioxidant activity of EBD.

**Figure 4:**
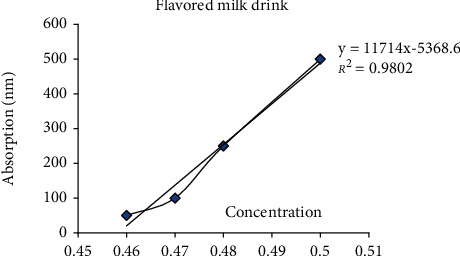
Antioxidant activity of FM.

**Figure 5:**
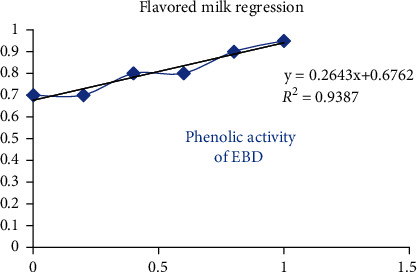
Quantitative phenols of the EBD.

The antioxidant test showed that as the concentration increases, there is increase in the value of reducing power (>0.9). The antioxidant activity of EBD showed that as the concentration increased, the absorbance also increased steadily (Figure 3). The antioxidant activity of FM showed that as the concentration increased, the absorbance also increased exponentially (Figure 4). The quantitative phenols of EBD slightly increased in absorbance on increasing concentration (Figure 5).

**Figure 6:**
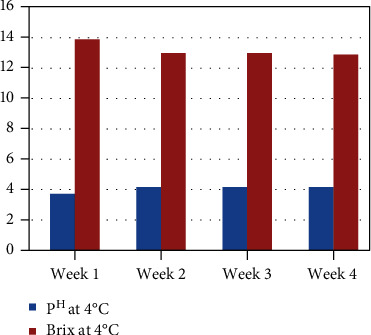
Product shelf life study (pH and brix) during storage of juice sample.

**Figure 7:**
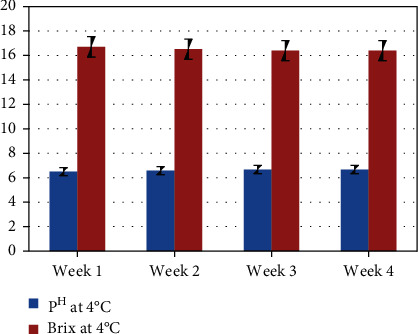
Product shelf life study (pH and brix) during storage of flavored milk.

The product shelf life study (pH and brix) during storage of juice sample was observed (3–4 pH) with high sugar content (11–15° brix) at weeks 1, 2, 3, and 4 (Figure 6). The product shelf life study (pH and brix) during storage of flavored milk was observed (4–7 pH) with a brix value of 13–26° at weeks 1, 2, 3, and 4 (Figure 7).

With regard to the descriptive sensorial analysis of flavored milk, the mean scores in trial 1 were 6.0 ± 0.00, 6.6 ± 0.55 (trial 2), 7.8 ± 0.45 (trial 3), 8.6 ± 0.55 (trial 4), and 8.8 ± 0.45 (trial 5) (Table 4). The statistical sensorial analysis of flavored milk reported a significant mean difference between groups 1 & 2, 1 & 3, 1 & 4, 1 & 5, 2 & 3, 2 & 4, 2 & 5, 3 & 4, and 3 & 5 with *p* < 0.0001 in between each group. An insignificant difference of *p* = 0.214 was observed in between groups 4 & 5 (Table 5). With regard to the descriptive sensorial analysis for EBD, the mean scores in trial 1 were 6.40 ± 0.55, 7.20 ± 0.45 (trial 2), 8.40 ± 0.55 (trial 3), 8.60 ± 0.55 (trial 4), and 8.80 ± 0.45 (trial 5) (Table 6). The statistical sensorial analysis for EBD reported a significant mean difference between groups 1 & 2, 1 & 3, 1 & 4, 1 & 5, 2 & 3, 2 & 4, and 2 & 5 with *p* < 0.0001 in between each group and *p* = 0.016 observed in 3 & 5. Insignificant differences of *p* = 0.255 and *p* = 0.214 were observed in between groups 3 & 4, and 4 & 5, respectively (Table 7).

## 4. Discussion

The present study is aimed at synthesizing Beetroot (Beta vulgaris) extract energy booster drink (EBD) and flavored milk (FM) followed by the analysis of its physicochemical properties and phytochemical constituents. Beetroot is a source of nutritional agents with possible application as a therapeutic agent for pathological ailments. The nutritional composition of beetroot (*Beta vulgaris*) per 100 g is observed to have a total energy content of 180 kJ (43 kcal), 9.56 g of carbohydrates, 1.61 g of proteins, 0.17 g of fats, 109 *μ*g folate, 0.067 mg of vitamin B6, and 4.9 mg of vitamin C [38, 39]. As compared with the above composition, in our study, the composition of energy booster drink (EBD) containing beetroot was 297.5 g/1000 ml for carbohydrates, 10.95 g/1000 ml for fats, 40.25 g/1000 ml for protein, and 29.2 ± 0.009 for fiber. The overall energy content was 352.27 kcal. This is higher than the total energy content of beetroot.

Energy drinks are a great source of carbohydrates and proteins, as there is a demand for energy drinks to provide nutritional supplement for optimum health. Conservative energy drinks have a large quantity of carbohydrates but a smaller amount of proteins [33]. However, organic energy drinks comprise of inadequate carbohydrates and a large amount of proteins and fibers, which include milk products with flavor manufactured by the extracts of beetroot [33]. In the present study, the constituent assessment between flavored and unflavored milk showed higher water, protein, lactose, and solid nonfat in flavored milk compared with unflavored milk. In addition, fat and protein contents in the flavored milk were 2.56% and 4.94%, respectively, in the present study. This is in contrast to other similar studies assessing beetroot-incorporated energy drinks, which showed 0.26% of fat and 1.12% protein content [40–42]. However, these studies formulated a beetroot mixture with pineapple and orange juice, in contrast with our study in which beetroot was mixed with milk. Therefore, the presence of milk increased the fat and protein contents in the present study.

In the present study, pH identification of the EBD and FM was performed as it is critical in maintaining the product properties. With reference to pH identification, a study by Nowak and Goslinski assessed two energy drinks wherein standard EDs were categorized by low pH ranging from 3.18 to 3.66 [43, 44]. The EDs enriched with fruits possessed slightly lower pH, i.e., from 2.32 to 3.60. The EDs having high acidity may possibly be affected mostly due to vitamin C [43, 44]. In the present study, energy drink had a more acidic pH, ranging from 3 to 4 than flavored milk (4––7 pH). This acidity of energy drink was attributed by the content of vitamin C, therefore, becoming more resistant to bacterial decomposition.

Multiple studies have highlighted health-related risks owing to a large amount of sugar in drinks (44–46). Clauson et al. [45] reported that a large quantity of sugar in EDs could risk the increase in obesity in populations. Other researchers have drawn attention towards the risks and hazards of increasing of type 2 diabetes mellitus and dental problems due to high-sugar energy drinks [46, 47]. Reddy et al. have stated that EDs hold a high quantity of free sugars, ranging from 25.5 g to 69.2 g, which, if taken regularly, can lead to dental erosion and the progression of obesity [48]. The present study indicated a moderate amount of sugar, i.e., 30 g in energy drink containing beetroot as well as in flavored milk. Therefore, this formulation of EBD and FM presents a low sugar and healthy alternative of beetroot-containing energy drinks.

It is suggested that sensory assessment of a developed product is significant for quality control and facilitates in worldwide trading of the product. In the present study, sensory scores for beetroot-flavored milk were assessed on the basis of attributes of color, aroma, taste, appearance, and the total acceptability, which showed scores of 6.00, 6.60, 7.80, 8.60, and 8.80 on the hedonic rating scale, respectively. In addition, majority of the groups showed a significant mean difference in sensorial analysis for both EBD and FM. Similar hedonic rating scale scores were observed in a study by Su et al., assessing the sensory score for pineapple-flavored milk on the basis of color, appearance, flavor, and taste [49]. In addition, a reduction in energy is a quantity of the attentiveness of combinations that are the donors of electron and can perform as primary and secondary elements of antioxidants [50]. Studies have reported that decreasing power increases as the sample concentration increases. Greater decreasing powers might be accredited to a greater amount of entire phenolic compounds and flavonoids, and the reducing power of a compound might reproduce its potential as an antioxidant [51]. Observations in the present study are consistent with the findings of earlier investigations indicating that the reducing power increased, as the sample concentration increased, suggesting the higher antioxidant potential of the compound along with the higher amounts of whole phenolic compounds and flavonoids in EBD compared with flavored milk.

Interestingly, in a study by Mensah et al., it was revealed that the plants that have had tannin; cardiac glycoside and alkaloid were most effective in improving cardiovascular function, reducing hypertension, and providing better support for heart function [52]. A similar study suggested that flavonoids are antioxidants preventing oxidative cells from impairment; they have strong anticancer elements as they provide protection against carcinogenesis at all stages [53, 54]. Additionally, plant-derived products were also found useful in promoting oral health and preventing dental diseases, i.e., periodontitis and dental caries, which are associated with systematic conditions like heart and joint problems [55, 56]. Interestingly, in the present study the presence of cardiac glycoside, flavonoids, and phytosterol in beetroot mixed with the milk as well as in EBD was observed. Therefore, the formulated EBD and FM present potential for antihypertensive and anticancer properties.

Although the present study reviewed on the beneficial properties of functional food on health, their outcomes on most of the diseases have not been completely explored and their functional activity for endorsing well-being and controlling illnesses must require additional investigation. Furthermore, another study reported on the health benefits of physical activity particularly in sports stating that dietary supplementation with it decreased (NO_3_) oxygen consumption at a submaximal level of exercise [57]. The study by Pima et al. [58] conducted on athletes was concluded after testing that they individually have a maximum volume of oxygen (VO_2_) and aerobic energy cost. Similarly, Wong et al. [59] reported that beetroot juice with supplementation will increase the athlete's performance during exercise. It was also emphasized that the cardioprotective effects of dietary nitrate from beetroot on healthy and hypertensive individuals are undeniable and irrefutable.

The analysis of bioactive components which are naturally present in the beetroot, through proteomic and genetic tests was not carried out in this study, due to a lack of resources. In the future, a study based on the characterization of biopeptide and bioactive components which are responsible to boost up the energy level or immunity is warranted on a large scale.

The study uniquely focused on the analysis of the beetroot supplement antioxidant capacity in energy booster drinks and flavored milk. The beetroot extract showed high-antioxidant components and was considered beneficial in hypertension, cancer, obesity, cardiovascular diseases therapy, and improved immune system. It shows that beetroot supplementation possesses potential and highly natural nutritional involvement in clinical sites.

## 5. Conclusion

This study concludes that energy drinks having beetroot indicated higher antioxidant components (total polyphenol concentration and antioxidant capacity) than flavored milk. The nutraceutical products (energy booster drink and flavored milk) containing beetroot are enriched with optimum quantities of proteins and fats and low carbohydrates at a stable pH with an adequate total energy content. Therefore, beetroot-based energy drink and flavored milk showed great potential due to high-antioxidant components and nutrients as a therapeutic in hypertension, cancer, obesity, cardiovascular diseases, and improved immune system.

## Figures and Tables

**Table 1:** Phytochemical screening of EBD & FM.

Chemical constituents	Tests	Results	
		Sample 1	Sample 2
Cardiac glycosides	Keller-Kiliani test	^**++**^	^**+++**^
Phytosterol test	Salkowski's test	^**++++**^	^**+++**^
	Liebermann-Burchard test	^**++++**^	^**+++**^
Phenolic compound	Ferric chloride test	^**++**^	**—**
	Lead acetate test	^**++**^	**—**
Flavonoid test	Alkaline reagent test	^**++**^	^**++**^
	Lead acetate test	^**++**^	^**++**^
	Ferric chloride test	^**+++**^	^**++**^
Terpenoids test	Salkowski's test	^**+++**^	^**++**^

^++^Good; ^**+++**^moderate; ^**++++**^strong; EBM: energy booster drink; FM: flavored milk.

**Table 2:** Qualitative composition of energy booster drink.

Composition of energy booster drink (EBD)	
Carbohydrate	297.5 g/1000 ml
Fat	10.95 g/1000 ml
Protein	40.25 g/1000 ml
Fiber	29.2 ± 0.009
Total energy: 352.27 kcal	

Kcal: kilocalorie; g: gram; ml: milliliter.

**Table 3:** Composition of flavored drink.

Composition of flavored Milk		Standard specification PS:3189-2012
Chemical tests		
Fat	2.56%	2%
Protein (FM)	4.94%	—
Protein (juice)	40.25 ± 0.09	—
Lactose	7.44%	—
SNF	13.4%	6%
Water	98.3%	—
Density	49.3%	—
Creaming index	19	20
Phosphatase test	Negative	Negative
Total energy 72.57 kcal		
Microbiological tests		
Total viable plate count (TVPC) CFU/ml/g	<10000 cfu/g	<50000 cfu/g
Salmonella	Absent	Absent/25g
Melamine	2.0 ppm	2.5 ppm
Aflatoxin (M_1_)	0.2 ppb	0.5 ppb

M1: standard test for Aflatoxin; SNF: solid not fat; CFU: colony-forming unit; FM: flavored milk.

**Table 4:** Descriptive sensorial analysis for flavored milk.

Variables	Trial 1	Trial 2	Trial 3	Trial 4	Trial 5
Color	6	7	8	9	9
Aroma	6	6	7	8	9
Taste	6	6	8	9	8
Appearance	6	7	8	8	9
Mean score	6.00	6.60	7.80	8.60	8.80
Standard deviation	0.00	0.55	0.45	0.55	0.45

**Table 5:** Statistical sensorial analysis for flavored milk.

Group	Mean diff.	*t*	df	*p* ≤ 0.05	Significance
1 & 2	−0.60	4.9	38	0.000	Yes
1 & 3	−1.80	18	38	0.000	Yes
1 & 4	−2.60	21.2	38	0.000	Yes
1 & 5	−2.80	28	38	0.000	Yes
2 & 3	−1.20	7.59	38	0.000	Yes
2 & 4	−2.00	11.5	38	0.000	Yes
2 & 5	−2.20	13.9	38	0.000	Yes
3 & 4	−0.80	5.06	38	0.000	Yes
3 & 5	−1.00	7.07	38	0.000	Yes
4 & 5	−0.20	1.26	38	0.214	No

**Table 6:** Descriptive analysis for energy booster drink.

Group	Trial 1	Trial 2	Trial 3	Trial 4	Trial 5
Color	7	7	9	9	9
Aroma	6	7	8	8	8
Taste	7	8	9	9	9
Appearance	6	7	8	9	9
Mean score	6.40	7.20	8.40	8.60	8.80
Standard deviation	0.55	0.45	0.55	0.55	0.45

**Table 7:** Statistical analysis for energy booster drink.

Group	Mean diff	*t*	df	*p* ≤ 0.05	Significance
1 & 2	−0.80	5.06	38	0.000	Yes
1 & 3	−2.00	11.5	38	0.000	Yes
1 & 4	−2.20	12.7	38	0.000	Yes
1 & 5	−2.40	15.2	38	0.000	Yes
2 & 3	−1.20	7.59	38	0.000	Yes
2 & 4	−1.40	8.85	38	0.000	Yes
2 & 5	−1.60	11.3	38	0.000	Yes
3 & 4	−0.20	1.15	38	0.255	No
3 & 5	−0.40	2.53	38	0.016	Yes
4 & 5	−0.20	1.26	38	0.214	No

## Data Availability

All data are available within the manuscript.
